# Supplementary material for: Complex‐centric proteome profiling by SEC‐SWATH‐MS
Source: Mol Syst Biol. 2019 Jan 14;15(1):e8438. doi: 10.15252/msb.20188438 (PMC6346213; doi:10.15252/msb.20188438)
Supplement: Supplementary file 7 — Dataset EV6 [file MSB-15-e8438-s007.zip › feature_plots_bioplex/P26641.pdf]

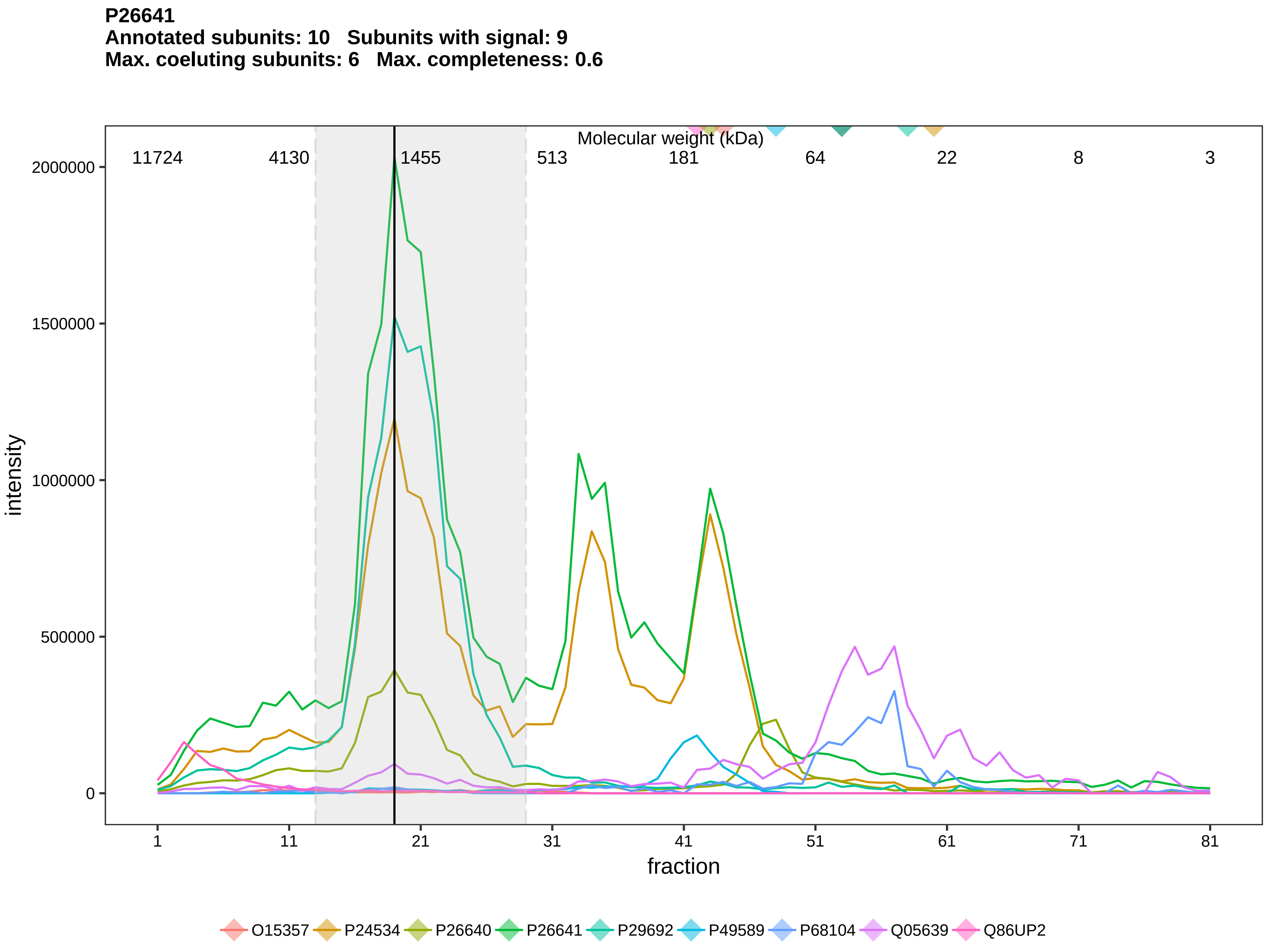
P26641
Annotated subunits: 10   Subunits with signal: 9
Max. coeluting subunits: 6   Max. completeness: 0.6
Molecular weight (kDa)
11724
4130
1455
513
181
64
22
8
3
intensity
2000000
1500000
1000000
500000
0
fraction
1
11
21
31
41
51
61
71
81
O15357
P24534
P26640
P26641
P29692
P49589
P68104
Q05639
Q86UP2
